# Fine Display of Chemicals, Etc.

**Published:** 1886-01-20

**Authors:** 


FINE DISPLAY OF CHEMICALS, ETC.—In answer to a kind invitation of Dr. J. W. Stone, representing the house of Parke, Davis & Co., we visited him on the 9th instant, at his room, 211 Kimball House, where we found a superb display of Drugs, Chemicals, and Modern Appliances, prepared by that celebrated firm. We will not attempt to give any description of what we saw, or of what we heard ; for let it be said, *en passant*, that we met there a large crowd of admiring visitors. We take this method of tendering our thanks to Dr. Stone for his courteous attention.

